# *Cladosporium cladosporioides*, endophyte of *Strelitzia nicolai*, as a new producer of Alternariol monomethyl ether with a potential cytotoxic activity

**DOI:** 10.1038/s41598-025-33343-6

**Published:** 2026-01-12

**Authors:** Nourhan M. Farag, Ashraf S. A. El-Sayed, Eman Fikry, Nora Tawfeek, Azza M. El-Shafae, Maher M. El-Domiaty

**Affiliations:** 1 Pharmacognosy Department, Faculty of Pharmacy, Zagazig University, Zagazig, 44519 Egypt; 2 Enzymology and Fungal Biotechnology Lab, Botany and Microbiology Department, Faculty of Science, Zagazig University, Zagazig, 44519 Egypt

**Keywords:** *Cladosporium cladosporioides*, *Strelitzia Nicolai*, Alternariol monomethyl ether, Anticancer activity, Topoisomerases inhibitors, Biochemistry, Biotechnology, Cancer, Chemical biology, Drug discovery, Microbiology

## Abstract

The emergence of chemotherapy resistance is one of the challenges in cancer therapy, for the adaptability of cells to shield the normal targets, and expressing of efflux pumps to the normal drugs. Thus, searching for lead compounds of multiple targets activity in tumor cells, from novel endophytic fungi of medicinal plants, could be a distinctive approach for cancer chemotherapy. *Cladosporium cladosporioides* EFBL-025*,* endophyte of *Strelitzia nicolai,* was recognized for the first time as a potential Alternariol monomethyl ether (AME) producer (700.1 μg/l). This isolate was morphologically, and molecularly identified based on the ITS with accession # PX463714. The chemical identity of AME of *C. cladosporioides* was committed from the FT-IR, HPLC and LC-MS/MS. The molecular mass of the parent molecule was 274.2 m/z, with the same molecular fragmentation pattern of authentic AME, as revealed from LC-MS/MS. AME has a significant activity against the A549 (IC_50_ 0.65 μg/ml), HepG-2 (IC_50_ 1.2 μg/ml), MCF-7 cells (IC_50_ 2.7 μg/ml), with selectivity index 28.2, 14.5 and 8.1 folds, respectively, compared to the OEC cells (IC_50_ 18.4 μg/ml). The activity of AME was a slightly higher than Taxol towards the tested cells by 1.8 folds. The purified AME has a higher inhibitory activity for Topoisomerase II (IC_50_ value 5.7 μg/ml) and I (IC_50_ value 7.25 μg/ml), compared to Etoposide (15.02 μg/ml) and Topotecan (27.27 μg/ml). The maximum arrest of the MCF-7 cells growth was observed at G0-G1 phase by 73.9 %, with a potency to induce the apoptosis by 12 folds compared to the control cells, suggesting the interference with the machinery of cellular growth and protein synthesis prior to DNA replication. Upon treatment of MCF-7 cells with AME, the healing activity was reduced by 12.5 % after 24. From the docking analysis, the binding energy of AME with the *αβ*-tubulin vinca site was -8.3 kcal/mol, compared to -7.4 kcal/mol for vinblastine. The AME binding affinities with Topoisomerase I, II*α* and II*β* were -7.3, -8.0, -8.3 kcal/mol, respectively, revealing the higher affinity for Topoisomerase II, as being consistent from the experimental analysis. Thus, this is the first reports exploring the metabolic potency of *C. cladosporioides* as AME producer, with the different cytotoxic insights.

**Supplementary Information:**

The online version contains supplementary material available at 10.1038/s41598-025-33343-6.

## Introduction

Chemotherapy is one of the profound approaches in cancer treatment, targeting a specific metabolic cellular machinery of the rapidly dividing cells^1^. According to their mode of action, four categories of the chemotherapeutic agents were recognized as follows; 1- Alkylating agents that interfere with the DNA replication, forming cross-links within the DNA strands, leading to apoptosis. 2-Antimetabolites, that inhibits the essential enzymes for DNA and RNA synthesis, subsequently disrupting the cell division. 3-Topoisomerase inhibitors, that prevent the DNA unwinding and replication via inhibiting topoisomerases, causing an ultimate DNA damage and cell death. 4- Mitotic inhibitors, that target the microtubules, preventing the proper chromosome segregation during mitosis, leading to cell cycle arrest^2^. Although, chemotherapy is one the most implemented tool in cancer prevention and treatment, however, the emergence of drug-resistance of tumor cells, systemic toxicity, especially with tumor heterogeneity, are the major challenges^3,4^. Thus searching for a novel lead compounds with a potential activity to target the different cellular machineries of the tumor cells is the challenges.

Fungi have been emerged as a repertoire of diverse secondary metabolites with potential biological activities^5^. Mycotoxins are one of the diverse secondary metabolites with various biological aspects ranging from toxic metabolites and carcinogenic to pharmaceutically important cytotoxic compounds^6–9^. *Alternaria* secondary metabolites are among those compounds with the preliminary cytotoxic activity particularly by topoisomerases inhibition^10^. The genus *Alternaria* produces a plethora of secondary metabolites namely alternariol, alternariol monomethyl-ether (AME), and tenuazonic acid (TeA) belongs to the dibenzo-pyrone derivatives, with diverse biological activities^11,12^. Alternariol (AOH) and alternariol monomethyl-ether (AME) (Djalonensone) are the common mycotoxins produced by the genus *Alternaria*^13^, that initiates the reactive oxygen species (ROS), with a higher affinity to interact with DNA topoisomerase I, and II, causing single and double strand breaks in DNA^13^. The presence of AOH and AME significantly decreases proliferation in mammalian cells, primarily due to cell cycle arrest, frequently occurring in the G2/M phase^10,14^. So, screening for a novel isolates with higher potency for AME production, as endophytes of various ethno-pharmaceutical medicinal plants could be an alternative strategy. The medicinal plants have been considered as repertoire of novel endophytic fungi of unique bioactive metabolites^15–17^. From the historical ethno-pharmaceutical background, the monocot family Strelitziaceae has been known with their richness of bioactive compounds^18^. Among the genera of Strelitziaceae, *Strelitzia nicolai*, that inhabits the tropical regions of the Southern Africa, possess a considerable pharmaceuticals of significance antioxidant and cytotoxic activity^19^, nevertheless, the endophytic fungi of this plant is poorly explored. Therefore, the objective of this study was to isolate and characterize potential endophytic fungal isolates from *S. nicolai* with the highest AME yield, chemical characterization of AME, exploring their mechanism of cytotoxicity, and molecular docking analysis.

## Materials and methods

### Plant sample collection, isolation of their endophytic fungi

The leaves samples of *Strelitzia nicolai* were collected from the Nursery at Shanbari, Oseem, Giza, Egypt in August 2024, identified by Dr. Therese Labib, Plant Taxonomist at Orman Botanical Garden, Egypt, and the samples were preserved as specimen voucher at the Pharmacognosy Department Herbarium of Zagazig University with deposition # ZU-Ph-Cog-801. The collected fresh leaves and petioles were transported in sterile plastic bags under aseptic conditions to the lab, washed with sterile distilled water prior isolation of their endophytic fungi. The leaves were sterilized by 70 % ethyl alcohol for 2 min, then 2.5 % sodium hypochlorite for 1 min, rinsing twice with distilled water, sectioned aseptically, and plated on potato dextrose agar (PDA) media with cefotaxime (1 mg/ml), incubated for 15 days at 30°C^15^. The emerged fungal colonies were collected and subcultured on the same media, and preserved as slope cultures at 4°C for the subsequent screening and analyses. The fungal colonies were identified to the species level based on their macro and micromorphological features, according to the universal fungal identification keys^16^.

### Antifungal activity guided-assay, and screening for the AME-producing endophytic fungi of S. nicolai

Production of the bioactive metabolites by the isolated endophytic fungi of *S. nicolai* were evaluated by culturing on potato dextrose broth (PDB) medium (200 g potato extract and 20 g glucose per liter)^5^. Two plugs of 6-day-old PDA culture of each isolate were inoculated into 50 ml of PDB medium/250 ml Erlenmeyer conical flask, incubated for 14 days at 30°C. Three biological replicates were prepared for each fungus. The cultures were filtered, the filtrates were centrifuged at 5000 rpm, extracted with ethyl acetate (EA) for two times, the extracts were concentrated by rotary evaporator at 45°C, and the residues were dissolved in methanol and stored at 4°C^20^. The bioactivity of crude EA extract was checked against *Aspergillus niger*, *Fusarium oxysporum*, *F. solani,* and *Cladosporium herbarum* as a model fungal pathogen^21^.

Different concentrations of the ethyl acetate of *C. cladosporioides* (5, 10, 15, 20 μg/ml) at 100 μl was amended to the 6 h pre-cultures of *A. niger*, *F. oxysporum*, *F. solani* and *C. herbarum* grown on PDA by well-diffusion method, the cultures were incubated for 8 days at 30°C, and the diameter of the inhibition zones were measured. Since, the biological activity has been used as a remarkable marker for the presence of bioactive metabolites^22,23^, the active crude EA extracts were further checked by the TLC. The EA extract was fractionated by the TLC (Pre-coated silica gel 60 F254) with methanol/ water of 80:20 (v/v), compared to authentic AME (Cat.# HY-W013863). The TLC plate was illuminated at λ_254_ nm, the spots gave the identical color and mobility rate of the authentic AME were considered^22,24,25^.

### HPLC, FT-IR, H NMR, and LC-MS/MS analyses

The spots of AME were extracted from TLC silica gel by scraping-off the target spots, dissolving in methanol^17,26^. The concentration of the extracted AME were assessed by HPLC (YOUNG In), with RP-C18 column (Cat.#. 959963-902). The mobile phase was methanol/ water at 80:20 v/v, with flow rate 1.0 ml/min at λ_365_ nm by UV-detector (YL910.UV-Vis, YOUNG In, chromass). The sample concentration was verified based on the retention time and peak area, compared to the authentic AME (Cat. #HY-W013863) at λ_365_ nm. The functional groups of putative AME were analyzed by the FT-IR spectrometer at 400-4000 cm^−1^, utilizing the KBr pellets.

The identity of putative AME was resolved by LC-MS/MS with a LCQ Deca mass spectrometer, in a positive ion mode electrospray source. A gradient elution of mobile phase (acetonitrile with 0.1% formic acid) was used from 2 to 98% at flow rate 0.2 ml/min, for 30 min. The identification of the chemical signals was based on their retention times and mass spectral fragmentation patterns, in comparison to authentic AME. The parent molecule of AME was undergoes MS/MS, and the molecular fragmentation pattern of the sample was compared to authentic one.

The chemical structure of purified AME was confirmed from the 1H NMR analysis, the samples were dissolved in CDCl3, the chemical shifts and coupling constants were represented in ppm (δ-scale) and hertz (Hz), respectively.

### Molecular identification of isolated endophytic fungi

The selected AME producing fungal isolates were identified based on the sequence of ITS domain^27,28^, and β-tubulin A (*ben A*) gene. The fungal genomic DNA (gDNA) was extracted by CTAB buffer^29^ and used template for PCR with the primer sets: ITS5 5ʹ-TCCTCCGCTTA-TTGATATGC-3ʹ, ITS4 5ʹ-GAAGTAAAAGTCGTAACAAGG-3ʹ^28^, and *ben* A F 5ʹ-GGTAAC-CAAATCGGTGCTGCT-TT-3ʹ and *ben* A R 5ʹ-ACCCTCAGTGTAGTGA-CCCTTGGC-3ʹ. The reaction contains 10 µl 2× PCR master mixture (Cat.#25-027), 1 µl gDNA, and 10 pmol of primers, adjusted to 20 µl final volume with distilled water. The PCR conditions were adjusted as described in our previous studies (El-Sayed et al., 2021, 2022). The PCR products were examined by 1.5 % agarose gel in 1 × TBE buffer^30^, purified, sequenced, the sequences were non-redundant BLAST searched on NCBI, aligned with the Clustal W muscle algorithm, and the phylogeny analysis was constructed by neighbor-joining method by MEGA X software^30–32^.

### Kinetics of AME production by C. cladosporioides with the incubation time, and the effect of S. nicolai extracts on the AME yield

The yield of AME by *C. cladosporioides* was assessed by incubating the fungal cultures grown on PDB, for 20 days at 30°C, then the cultures were filtered intervally and extracted with ethylacetate, as described above. The ethylacetate extract was concentrated by rotary-evaporator, and checked by the TLC, compared to the authentic AME. The putative spots of AME were extracted, and the purity and concentration of AME was assessed by HPLC, normalized to authentic AME, as described above.

The influence of methanolic extracts of *S. nicolai* on the AME yield by *C. cladosporioides* was assessed. The fresh leaves (20 g) of *S. nicolai* were minced in 50 ml of absolute methanol, and the mixture was stored at 4 ℃ for 12 h at 200 rpm agitation, and the resulting extracts were filtered, centrifuged at 5000 rpm for 10 min, and the extracts were dried, and amended at 0.05, 0.1 and 0.4 g /50 ml PDB of 5 day-old cultures of *C. cladosporioides.* The fungal cultures were incubated at standard conditions for 7 days, then, the cultures were filtered, and extracted with ethylacetate, and the extracts was fractionated by TLC, as described above. The putative spots of AME from the TLC were extracted, and the concentration and purity of the target compound was assessed by HPLC, as described above.

### Antiproliferative activity of purified AME

The activity of purified AME from the potent fungus against the human hepatocellular (Hep-G2), breast (MCF-7), lung (A549) carcinoma and Olfactory ensheathing cells (OEC cells) was evaluated by the MTT assay^33,34^. The human HepG-2 (ATCC HB-8065), A549 (ATCC CCL-185), and MCF7 (ATCC HTB-22) cell lines were obtained from the American Type Culture Collection. The cells were cultured on DMEM (Invitrogen/Life Technol.) supplemented with 10% FBS (Hyclone), 10 μg/ml of insulin (Sigma), and 50 U/ml penicillin and 50 μg/ml streptomycin. The microtiter plate was inoculated by 2 x 10^3^ cells/well, incubated for 12 h at 37 °C, then AME was amended with different concentrations, and the plate was incubated for 48 h at the same conditions. The MTT reagent was added to the wells, incubated for 6 h, and the resulting purple compound was measured at λ_570_ nm. The IC_50_ and CC_50_ values were determined as the drug concentration decreases the initial cellular growth by 50% for the tumor cells and control cells, respectively^33^. The selectivity index was expressed by the CC_50_ value of OEC cells to the IC_50_ value of tumor cells^35^.

### Kinetics of inhibition of DNA Topoisomerase I and II by the extracted AME

The activities of topoisomerases I and II were determined according to the Kits instructions (Topogen Cat. #. 1015-1 and Cat. #. TG1001-1), by converting the supercoiled circular DNA to relaxed DNA. For Topoisomerase I, the reaction mixture contains supercoiled DNA, 10x assay buffer, and enzyme, in presence of different AME concentrations. The reaction was incubated for 30 min at 37°C, checked by 1% agarose gel, the DNA was visualized, and the value of IC_50_ was expressed by AME dose inhibiting the activity of topoisomerases by 50 % compared to control. The topoisomerase II was assessed by the release of mini-circular DNAs by decatenation of the intertangled mass of kDNA, by binding robustly to kDNA networks, thus, rapidly releasing intact 2.5 KB monomeric rings that rapidly migrate to the 1 % agarose gels, while, the extremely large mass of DNA fails to enter a 1% agarose gel. The reaction mixture contains 5 x reaction buffer, kDNA, topoisomerase II, and different concentrations of AME in 20 μl total reaction volume by distilled water, the mixture was incubated for 30 min, at 37°C, stopped by 5X stopping buffer, and the relaxed or minicircular DNA was assessed by 1% agarose gel.

### Tubulin polymerization inhibition by the AME

The activity of *C. cladosporioides* AME to inhibits tubulin polymerization was evaluated by fluorescence assay (Cat.# BK011P)^36^. Heterodimer tubulin is a protein of 55 kDa (α and β tubulins), from porcine brain tissue was used as highly homologous to tubulin of mammalian cells. Tubulin protein (4.0 mg/ml) was suspended in 500 μl Polymerization Buffer (80 mM PIPES (pH 6.9), 2 mM MgCl_2_, 0.5 mM EDTA, and 1.0 mM GTP), added to the wells of 96-well plate of different doses of the AME. Positive control of Paclitaxel was used. The reaction mixture was incubated for 15 min, and the tubulin polymerization was determined by checking the turbidity at λ_340_nm (VersaMax™).

### Apoptosis, cell cycle analysis of MCF-7 cells for the purified AME

The apoptosis process of MCF-7 cells was determined by the Annexin-Apoptosis assay Kit (Cat.#. K101-25) that based on the externalization of phosphatidylserine (PS) to the cell surface during apoptosis, and the developed Annexin V-PS complex was quantified by flow cytometry^30^. The cells (2 × 10^5^ cell/well) were inoculated to the 96-well plate, amended with the AME at IC_50_ value, incubated for 24 h. The cultures were collected, rinsed with 1 ml of PBS, followed by addition of 200 μl of 1X Annexin-binding buffer, and Annexin V-FITC and PI to the cells, and incubated in dark for 15 min. Annexin-PS complex was then identified using a FITC signal detector at (Ex, λ_488_ nm; Em, λ_530_ nm).

The cell cycle of MCF-7 for the AME was evaluated by propidium iodide (PI) assay (Cat #. ab139418). The plate were seeded with the cells, incubated overnight at 37°C, followed by adding AME at the IC_25_ value, and re-incubated for 48 h. The plat culture was centrifuged at 2000 rpm for 5 min, fixed in ice-cold ethanol for 2 h at 4°C. The cells were rehydrated with PBS, stained with a propidium iodide solution for 30 min in dark, and the DNA contents was assessed by flow cytometry at Ex λ_493_ nm and Em λ_636_ nm, to calculate the percentages of G0/G1, S, and G2/M phases^22,26^.

### Anti-wound healing activity of purified AME

The influence of purified *C. cladosporioides* AME on ceasing the migration of the MCF-7 tumor cells was evaluated. The 24-wells microtiter plate were seeded with 5 × 10^6^ cells/well, and permitted to grow as confluent monolayer, a wound was made, washed with PBS, and amended with fresh medium of AME at its IC_25_ value. The culture was incubated at 37 °C at 5% CO_2_, and the wound closure was imaged by phase-contrast microscope. The wound healing was expressed by the area of scratch closure in drug-treated cells, compared to untreated cells^26^.

### Molecular docking analysis

The molecular docking was conducted to assess the binding affinity and intermolecular interactions of AME with the specific tubulin and topoisomerase sites. Structural templates were used from the Protein Data Bank, including tubulin binding domains: the β-Tubulin Taxane Site (ID: 1JFF)^37^, β-Tubulin Colchicine Site (ID: 1SA0)^38^, and αβ-Tubulin Vinca Site (ID: 7Z7D)^39^; as well as topoisomerases targets: Topoisomerase I (ID: 1EJ9)^40^, Topoisomerase IIα (ID: 5GWK)^41^, and Topoisomerase IIβ (ID: 3QX3)^42^, that were accessed via RCSB PDB^43^. Protein preparation involved hydrogen addition, charge assignment, and energy minimization using BIOVIA Discovery Studio v21.1.0.20298^44^ and UCSF Chimera 1.17.3^45,46^. Active site prediction was conducted with the CASTp server^47^, with grid coordinates derived from the identified binding pockets. The 3D structure of the AME was obtained from PubChem, energy minimized, converted to the pdbqt format using OpenBabel 2.4.1^48^. Docking simulations were carried out by AutoDock Vina 1.1.2 integrated within UCSF Chimera, employing default parameters to generate 10 conformations per run, with binding affinities reported in kcal/mol (lower values indicate stronger binding). The optimal pose was selected based on the lowest root mean square deviation (RMSD) relative to co-crystallized ligand. Protein–ligand interactions were subsequently visualized, and analyzed by BIOVIA Discovery Studio Visualizer v21.1.0.20298.

### Statistical analysis

Three biological replicates were conducted for each experiment, and the yield of AME was expressed by means ± STDEV. The *p*-value was assessed by one-way ANOVA.

### Fungal deposition

The ITS sequence of *C. cladosporioides,* an endophyte of *S. nicolai,* was deposited to Genbank with accession # PX463714. The isolate was physically deposited to Enzymology and Fungal Biotechnology Lab, Zagazig University, Egypt with deposition # EFBL-NM-025.

## Results

### Isolation, and screening for the AME-producing fungi inhabiting *S. nicolai*

Twenty-four endophytic fungal isolates were isolated from the leaves and petioles of *S. nicolai* on PDA and malt extract agar media. These isolates were identified based on their microscopical features that belong to four genera *Cladosporium*, *Aspergillus, Fusarium* and *Penicillium* as described in Table S1. The recovered isolates were grown on PDA media, then their extracellular metabolites were extracted with ethylacetate (EA) and checked by TLC, and the putative concentration was calculated by ImagJ software package. From the screening analysis of the twenty-four isolates (Table S1), the EA extract of *Cladosporium* sp EFBL-025 had the highest intensity of the same colored spots and mobility rate of the authentic AME (Cat.# HY-W013863), as visualized at λ_254_ nm, with the approximated amount of AME (450 μg/l), followed by *Cladosporium* sp EFBL-28 (240 μg/l), *Alternaria* EFBL-32 (202 μg/l), and *Drechslera* sp EFBL-40 (150 μg/l). While, the other EA extracts of the recovered fungal isolates didn’t had any robust matched spots with the authentic AME, negating the presence of AME on their extracts. The biological activity of the recovered fungal extracts was assessed towards *A. niger, A. fumigatus,* and *F. oxysporum* as a model human pathogen. Among the recovered fungal isolates, the isolate EFBL-025 had an obvious antifungal effect against the tested model fungi in a concentration dependent pattern. The diameter of the inhibition zones of the EA extract of isolate EFBL-025 towards *A. niger, A. fumigatus* and *F. oxysporum* and *C. herbarum* were 4.2 mm, 6.4 mm, 7.0 and 10 mm at 20 μg/ml (Fig. S1). However, the remaining fungal isolates didn’t display any obvious biological activity against the tested fungi, compared to EA extract of control media. The biological activity guided-assay has been profoundly used as a preliminary sign to the presence of cytotoxic compounds. So, from the TLC semi-quantitative analysis and biological activity-driven assay, the EA extracts of isolate *Cladosporium* sp EFB-025 had the most antifungal activity, with a potential producing potency to AME.

### Morphological, molecular identification of the promising AME-producing fungi

The identity of *Cladosporium* sp EFBL-025 was determined relied on its macro and micro-morphological features^49,50^. The colonies were appeared as olive-green to olive-brown, with velvety appearance on PDA, with olive-grey to white, feathery edges, with diffused colonies in agar. The conidiophores are cylindrical, arising from immersed/swollen hyphal cells, with a single terminal conidiogenous cell, conidia of 2-5 μm wide and 0-1-septate, the secondary ramoconidia usually aseptate. These morphological features of the most potent AME producing isolate *Cladosporium* sp EFB-025 are typically follow those of *C. cladosporioides*^49,50^.

**Fig. 1:**
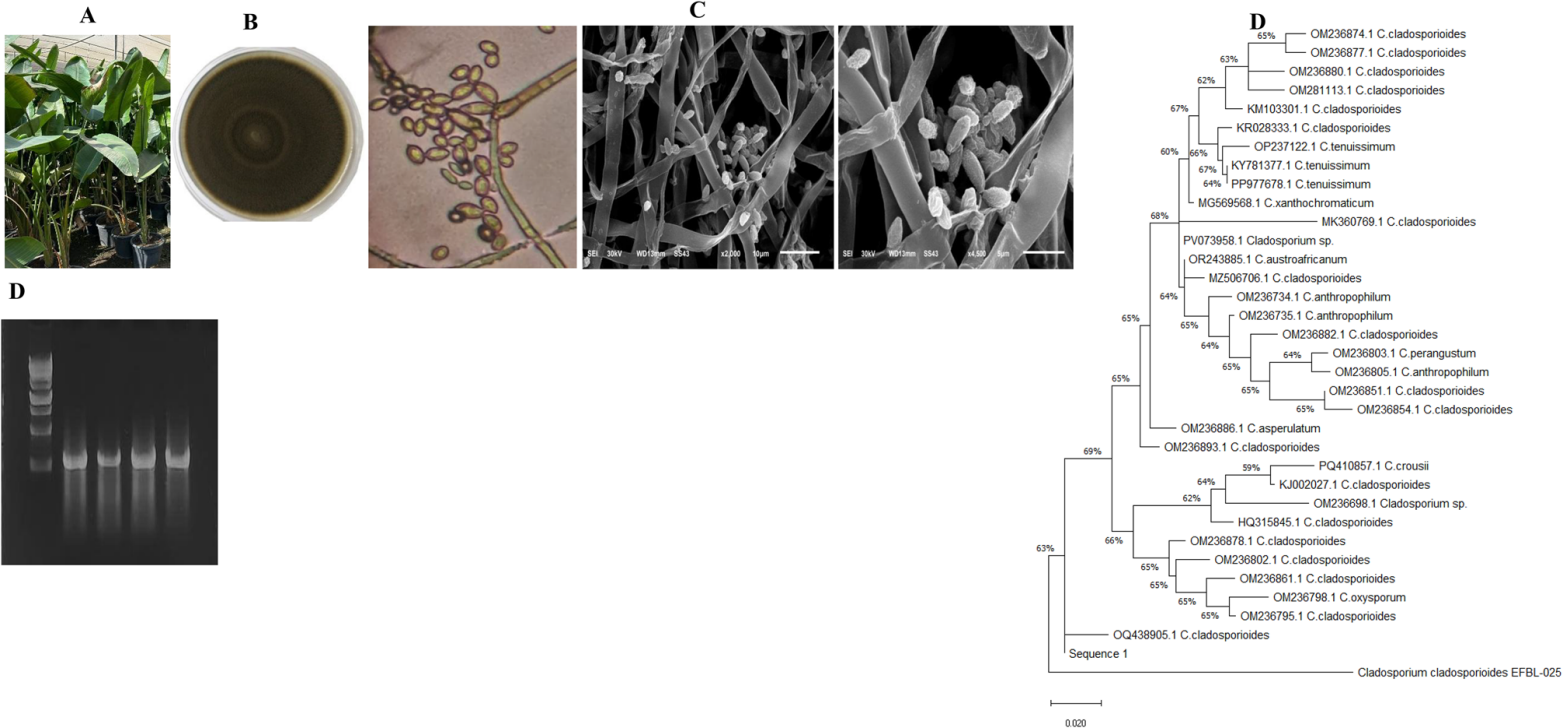
Morphological and molecular identification of the most potent AME producing fungal isolate inhabiting *Strelitzia nicolai.* (**A**) Morphological view of *S. nicolai.* (**B**) Plate culture of the potent fungal isolate. (**C**) Conidial ontology of the fungus at 1000, 2000, 4500 X. (**D**) PCR amplicons of the ITS region of *Cladosporium cladosporioides.* (**E**) Phylogenetic tree of the ITS sequences of the fungal isolate by Maximum Likelihood method with MEGA X software package.

The morphological identity of *C. cladosporioides* EFBL-025, an endophyte of *S. nicolai,* as the most potent AME-producer was verified based on its ITS sequence of the rDNA region. Using the gDNA as a PCR template, the ITS amplicon was resolved as 700 bp on 1.5% agarose gel (Fig. 1). The ITS sequence was non-redundantly BLAST searched on the gene bank of the NCBI database. The phylogenetic relatedness of the ITS sequence of the current isolate was constructed by the Maximum likelihood by the MEGAX software package (Fig. 1). From the phylogenetic analysis, the target ITS sequence had a 99.5 % similarity with the ITS sequences of *C. cladosporioides* OQ438905.1, OM236795.1, OM236798.1, OM23686.1, OM236878.1, HQ315845.1, OM236698.1, KJ002027.1, OM236893.1 and OM236851.1 with query coverage 100 % and zero E-value. The ITS sequence of the *C. cladosporioides* EFB-025 has been deposited on the gene bank with accession # PX463714. Thus, from morphological features and molecular analysis, the isolate was verified as *C. cladosporioides.*

### Chemical analysis of the putative AME

**Fig. 2:**
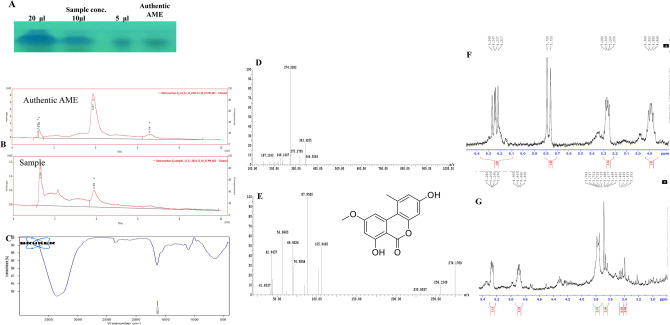
Chromatographic and spectroscopic analysis of the purified AME from *C. cladosporioides*. After incubation of the fungal cultures, the ethyl acetate extract of the fungal cultures was dried, fractionated by TLC analysis (**A**), their purity were checked by HPLC (**B**), their structural-functional groups were determined by FT-IR (**C**). E, The molecular mass of the parent molecule of AME by LC/MS (274.2 m/z) (**D**), and their molecular fragmentation pattern by LC-MS/MS were determined (**E**).

The EA extract of the most active AME-producing isolate “*C. cladosporioides*” was fractionated on semi-preparative TLC, and the assumed spots of AME with the identical color and mobility rate of authentic one was scraped-off, dissolved in methanol, and used for AME extraction. The efficiency of extraction was rechecked by TLC, the concentration of extracted AME was confirmed by the HPLC. From the HPLC chromatogram (Fig. 2), the purified AME sample of *C. cladosporioides* had a sharp peak at 3.8 min, that being identical to authentic AME (3.8 min). The UV-absorption spectral analysis revealed that the purified AME showed a maximum peak at λ_365_ nm, aligning with the absorption of the authentic AME. The functional groups of purified AME of *C. cladosporioides* were determined from the FTIR. The sample had a peak at 3430 to 3350 cm^-1^ that refers to the stretching of hydroxyl, and amide groups. Additionally, a stretch of the aliphatic CH groups in range of 2935 to 2855 cm^-1^, was observed. The peak at 1635 cm^-1^ was designated to C=O groups and aromatic rings stretch.

The molecular mass of purified AME of *C. cladosporioides* was determined by the LC-MS/MS as 274.2 m/z, as shown in Fig. 2. Furthermore, the parent molecule of 274.2 *m/z* was fragmented giving molecular ion peaks at 256.12, 105.9, 87.9, 70.9, 69.9, 56.9, 42.9 and 41.83 m/z, that being closely matched with the fragmentation pattern of authentic AME, ensuring the chemical structure of the purified sample as AME.

The chemical structure of purified AME of *C. cladosporioides* was confirmed by 1H NMR spectra. The resolved signals of 1HNMR for fungal extracted AME samples were identical to authentic one, 2.74 (3H, s, CH_3_), 3.78 (3H, s, CH_3_O), 6.21 (1H, d), 6.23 (1H, d), 6.24 (1H, d), 6.26 (1H, d), as consistently with the HNMR pattern of AME of *Alternaria* sp, an endophyte of *Salvia miltiorrhiza*^51^. Thus, from the TLC, HPLC, FT-IR, HNMR and LC-MS/MS, the chemical identity of the purified sample was confirmed as AME.

### The AME yield with the incubation time and effect of host plant extracts on its biosynthetic stability by C. cladosporioides

**Fig. 3:**
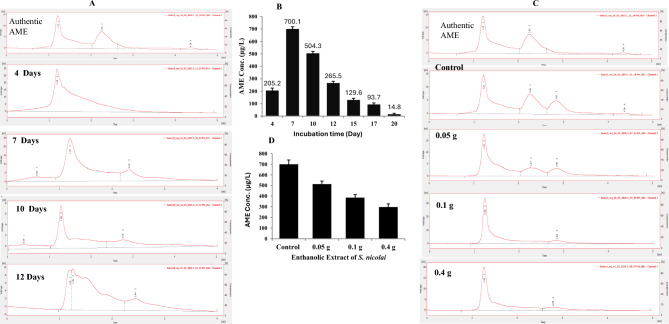
Kinetics of AME production by *C. cladosporioides* with the different incubation time (4, 7, 10 and 12 days), and in addition to amendment with ethanolic extracts of *S. nicolai*. After incubation, the cultures were extracted with ethyl acetate, and the fractionated by TLC, purified and quantified by HPLC. (**A**) HPLC chromatograms of the TLC-purified AME from *C. cladosporioides* cultures, incubated at different times*.* (**B**) Overall concentration of AME by *C. cladosporioides* with the incubation period. (**C**) HPLC chromatogram of the AME of *C. cladosporioides* amended with different concentration of ethanolic extracts of *S. nicolai* . (**D**) The AME concentration by *C. cladosporioides* in response to amendment with extracts of *S. nicolai.*

The influence of incubation period of *C. cladosporioides* on their AME concentration was studied intervally along 20 days, at standard conditions. After the desired incubation time, the cultures were filtered, the filtrate was centrifuged at 5000 rpm, extracted by ethylacetate, and checked by the TLC, compared to authentic AME. From the TLC, the yield of AME was significantly increased at the 7^th^ days of incubation (*p*-value <0.05), followed by a gradual decrease to the intensity of the putative AME spots with the further incubation days, compared to the authentic AME. The concentration of AME from *C. cladosporioides* was further validated by the HPLC (Fig. 3 A, B), the maximum yield of AME was reported after 7^th^ days of incubation (700.1 μg/l), followed by 10^th^ day (504.3 μg/l), and 12^th^ day (265.5 μg/l), as revealed from the peak area compared to authentic AME, at retention time 2.7 min. Thus, from the semi-quantitative TLC and quantitative HPLC, the highest yield of AME was obtained after 7^th^ days of incubation (700.1 μg/l), with a dramatic suppression to the AME yield at 20 days of incubation.

In an endeavor to assess the effect of methanolic extracts of the host plant *S. nicolai* on inducing the yield of AME by *C. cladosporioides,* different methanolic extracts was amended to the fungal cultures, and the concentration of AME was measured by TLC and HPLC. From the HPLC profile (Fig. 3C, D), the methanolic extract of *S. nicolai* has no positive effect on inducing the AME yield by *C. cladosporioides,* negating the presence of any metabolites derived from the host plant inducing the biosynthetic machinery of AME.

The yield of AME by *C. cladosporioides* was checked by storage of the first isolated cultures as plate culture at 4°C, then measuring their AME yield monthly for 10 months. Practically, the yield of AME by the *C. cladosporioides* was quietly stable along the tested 10 months, as revealed from the TLC and HPLC, ensuring the relevant biosynthetic stability of AME by *C. cladosporioides,* independent on external stimuli from plant (Data not shown)*.*

Prior to the cytotoxic analyses, the target compound was re-purified by preparative TLC, and checked again by the HPLC. After the final purification step, the yield of purified AME for the subsequent cytotoxic analysis was 500 μg/l with the total purity 85%. The purity of the compound was calculated for the percentage of the area of the main peak/ sum of area of main peak and other peaks.

### Antiproliferative, Topoisomerases I, II inhibition, and anti-tubulin polymerizing activities of C. cladosporioides AME

**Fig. 4:**
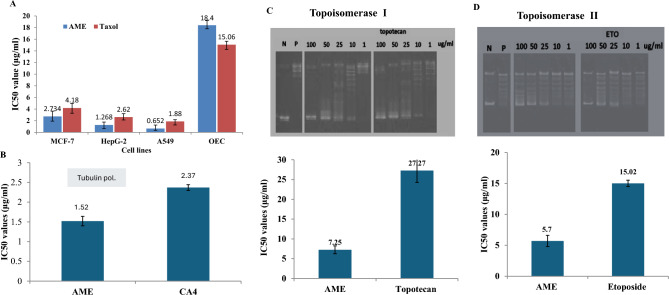
Antiproliferative, anti-tubulin polymerizing, and anti-Topoisomerase I and II activities of the purified *C. cladosporioides.* (**A**) The IC50 values of purified AME against the MCF7, HepG-2 cells, A549 and normal OEC cells, compared to Taxol as a positive control. (**B**) Kinetics of Tubulin inhibition by the purified AME, normalized to CA4 as a reference drug. C, Kinetics of Topoisomerase I (**C**) and II (**A**) inhibition at different concentrations of *C. cladosporioides* AME*.*

The cytotoxic activity of the purified *C. cladosporioides* AME against the tumor cells “HepG-2, MCF-7, and A549” was evaluated, compared to the normal OEC cells. The compound was added to the RPMI growth medium of tumor cells, then the cells viability were assessed by MTT assay. The initial amount of purified AME was 500 μg/l with 85% purity. From the results, the viability of the cells was strongly reduced in a concentration-dependent manner by the compound*.* From the IC_50_ values (Fig. 4), the purified AME had the highest activity against A549 (0.65 μg/ml), HepG-2 (1.2 μg/ml), MCF-7 cell (2.7 μg/ml), compared to the OEC cells as control (18.4 μg/ml). Practically, the cytotoxic activity of the purified AME compound was slightly higher than Taxol, towards all the tested cell lines, by about 1.8 folds. The selectivity index of purified AME of *C. cladosporioides* for the A549, HepG-2, and MCF-7 cells was 28.23, 14.5, and 8.1 folds, respectively, normalized to OEC control cells.

The inhibitory effect of purified *C. cladosporioides* AME for topoisomerase I and II was assessed relied on the amount of the circular DNA to the relaxed ones. The mobility of the supercoiled and relaxed DNA in response to different concentration of AME, on agarose gel was shown in Fig. 4. From the kinetics of enzyme inhibition, the IC_50_ values of *C. cladosporioides* AME for topoisomerase I was 7.25 μg/ml, compared to 27.27 μg/ml of Topotecan, i.e the activity of AME to inhibit topoisomerase I was 3.7 folds higher than Topotecan. As well as, the IC_50_ value for AME towards topoisomerase II was 5.7 μg/ml, compared to Etoposide (15.02 μg/ml), i.e the AME had a higher inhibitory activity to topoisomerase II by 3 folds than Etoposide as authentic topoisomerase II inhibitor. From the kinetics of inhibition, the AME had a higher affinity as inhibitor to topoisomerase II than I by about 27 %.

### Cell cycle, apoptosis analyses and anti-wound healing activity of AME-treated MCF-7 cells

**Fig. 5:**
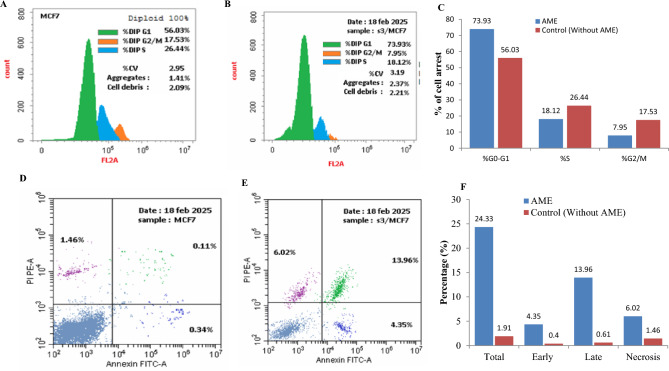
Cell cycle and apoptosis of the MCF-7 cells in response to AME *C. cladosporioides.* The cell cycle of MCF-7 cells in absence of AME (**A**) and in presence of AME (**B**) and the overall cellular arrest (**C**). The apoptotic analysis of the MCF-7 cells in absence of AME (**D**) treated with AME (**E**) of *C. cladosporioides* (**E**) and the overall apoptotic ratios (**F**).

The cell cycle of MCF-7 cells in response to *C. cladosporioides* AME at their IC_25_ value (1.3 μg/ml) was analyzed. The MCF-7 cells were selected, as the most resistant ones to AME, than the other tested cells. After the cellular incubation, the G0-G1, S and G2-M percentage was assessed as shown in Fig. 5. From the results, the highest growth arrest was observed at the G0-G1 phase by 73.9 %, compared to 56.03 % of the control cells (without drug). At the S and G2/M phases, the percentage of cellular arrest of MCF-7 treated with AME was 18.12 % and 7.95%, compared to 26.44 % and 17.5% of control cells. From the cell cycle analysis, the AME of *C. cladosporioides* had an obvious inhibitory effect to the cell cycle at their G0-G1 phase, suggesting the interference with the machinery of cell grows and protein synthesis prior to DNA replication.

The potency of the purified putative *C. cladosporioides* AME to induce apoptosis of the MCF-7 cells was determined by Annexin V-PI assay. The AME had a significant effect on inducing the apoptosis processes of MCF-7 cells by about 12 folds compared to the control cells (Fig. 5). The compound triggers the early apoptosis, late apoptosis, and necrosis of MCF-7 cells by 4.35. 13.96, and 6.02%, compared to 0.4, 0.61 and 1.46 % of the control, respectively. Thus, upon treatment of the MCF-7 by AME, the early, late apoptosis, and necrosis were increased by 10.8, 22.8 and 4.12 folds, respectively, compared to control cells.

**Fig. 6:**
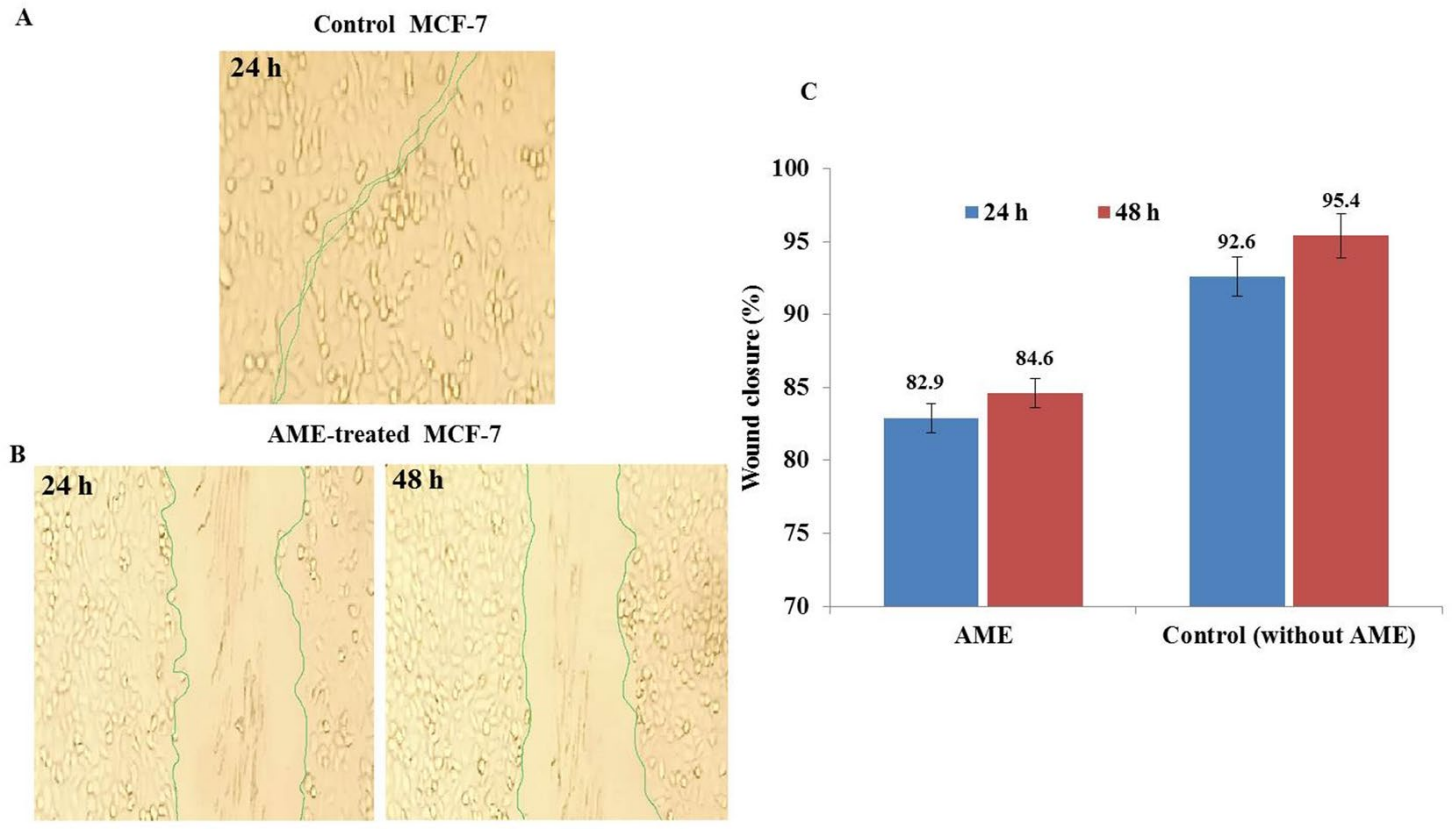
Anti-wound healing activity of the purified AME of *C. cladosporioides* towards the MCF-7 cells*.* A, Wound healing of MCF-7 cells at zero AME (**A**) and treated with *C. cladosporioides* AME at its IC_25_ value (**B**) after 24 and 48 h. C, The percentage of wound healing of the MCF-7 cells in response to AME *C. cladosporioides* , after 24 and 48 h, compared to control.

The anti-wound healing activity of the extracted *C. cladosporioides* AME on migration of MCF7 cells was assessed by quantifying the gap closure after 24, and 48 h, normalized to the control (Fig. 6). The wound healing activity of the MCF-7 cells in presence of AME was 82.9 and 84.6%, compared to the control cells of 92.6 and 95.4%, after 24 and 48h, respectively. So, upon treatment of MCF-7 cells with AME, the healing activity of cells was reduced by about 12.5 % after 24 and 48 h, compared to control.

### Molecular docking analysis of AME with tubulin and topoisomerase binding sites

**Fig. 7:**
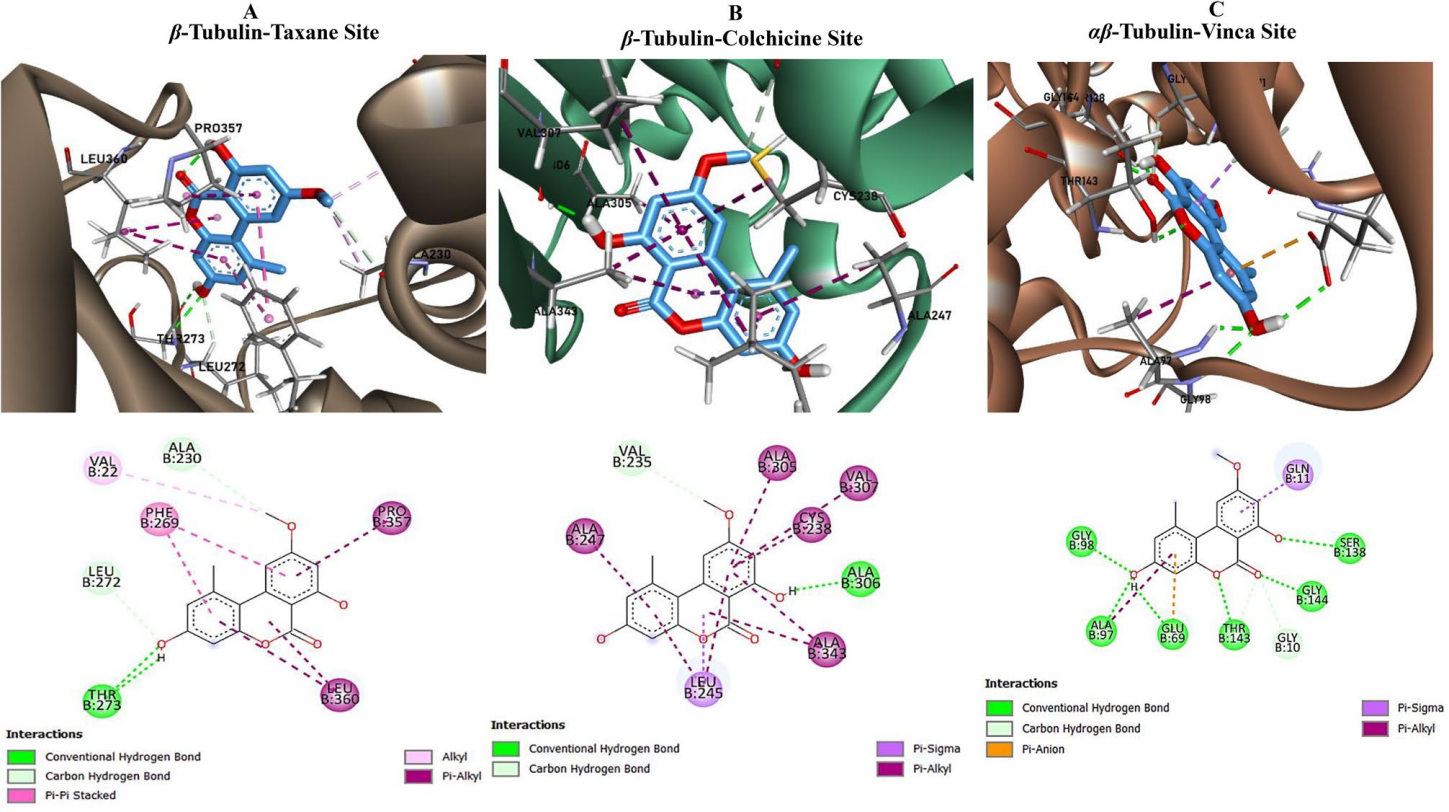
The three-dimensional (3D) (Upper panel) and two-dimensional (2D) (Lower panel) of β-tubulin-taxane (**A**) β-tubulin-colchicine (**B**) and β-tubulin-Vinca (**C**) sites docked with the AME. The interactions, and bond lengths of the AME with the experimented active sites of β-tubulin was illustrated from the 2D and 3D structures.

*In vitro* assays against *β*-tubulin and topoisomerases I and II revealed significant inhibitory activity by AME, which motivated further investigation into its binding interactions through molecular docking studies. The docking simulations were performed across several protein binding sites as Tubulin Binding Sites and Topoisomerases sites as summarized in Table 1. The AME displayed a binding affinity of –7.1 kcal/mol with the *β*-tubulin taxane site, in contrast to Taxol’s -9.9 kcal/mol. Analysis of the interactions (Fig. 7), revealed alkyl bonding with VAL22, carbon hydrogen bonds with ALA230 and LEU272, two *π*–*π* stacking interactions with PHE269, two conventional hydrogen bonds with THR273, and *π*–alkyl interactions with LEU360 and PRO357. At *β*-tubulin colchicine site, the AME achieved an affinity of -8.2 kcal/mol, slightly exceeding the binding affinity observed for colchicine (–8.1 kcal/mol) (Table S2). The ligand engaged in a conventional hydrogen bond with ALA306, a carbon hydrogen bond with VAL235, a *π*–sigma interaction with LEU245, and multiple *π*–alkyl contacts involving residues such as ALA247, ALA305, VAL307, and CYS238 (Fig. 7). At *αβ*-tubulin vinca site, AME recorded an affinity of –8.3 kcal/mol, compared to –7.4 kcal/mol for vinblastine. Binding at this site was mediated by conventional hydrogen bonds with GLU69, ALA97, GLY98, THR143, SER138, and GLY144, supplemented by carbon hydrogen bonds with GLY10 and THR143, a *π*–anion interaction with GLU69, a *π*–sigma bond with GLN11, and a π–alkyl interaction with ALA97 (Fig. 7).

**Table 1:** Docking results and interaction profiles of AME with the *β*-Tubulin and Topoisomerases active sites.

Target	Ligand	Binding affinity (kcal/mol)	Key interactions (AME)
*β*-Tubulin (Taxane Site)	**AME**	**-7.1**	**Alkyl:** VAL22; **Carbon-hydrogen:** ALA230, LEU272; **π–π stacking:** PHE269 (two bonds); **Conventional hydrogen:** THR273 (two); ***π*–alkyl:** LEU360, PRO357
	**Taxol co-ligand**	**-9.9**	-
*β*-Tubulin (Colchicine Site)	**AME**	**-8.2**	**Conventional hydrogen:** ALA306; **Carbon-hydrogen:** VAL235; **π–sigma:** LEU245; **π–alkyl:** ALA247, ALA305, VAL307, CYS238, LEU245 (two), ALA343 (two)
	**Colchicine co-ligand**	**-8.1**	-
*αβ*-Tubulin (Vinca Site)	**AME**	**-8.3**	**Conventional hydrogen:** GLU69, ALA97, GLY98, THR143, SER138, GLY144; **Carbon-hydrogen:** GLY10, THR143**; π–anion:** GLU69; **π–sigma:** GLN11; **π–alkyl:** ALA97
	**Vinblastine co-ligand**	**-7.4**	-
Topoisomerase I	**AME**	**-7.3**	**Conventional hydrogen:** ARG162, LYS291, THR299; **π–donor hydrogen:** ASP331; π–cation: LYS291; **π–anion:** ASP331 (two); **π–sigma:** LYS330; **π–alkyl:** PHE159, ARG162
Topoisomerase II*α*	**AME**	**-8.0**	**Conventional hydrogen:** ARG292, TRP405, ASP566, GLY569; **Carbon-hydrogen:** LEU287; **Alkyl:** PRO289; **π–alkyl:** LEU394, PHE565
Topoisomerase II*β*	**AME**	**-8.3**	**Conventional hydrogen:** SER226, ARG236, GLU348, TRP349; **Carbon-hydrogen:** TRP349, GLY516; **π–π stacking:** PHE512 (two); **π–sigma:** PHE512; **π–alkyl:** PRO233

**Fig. 8:**
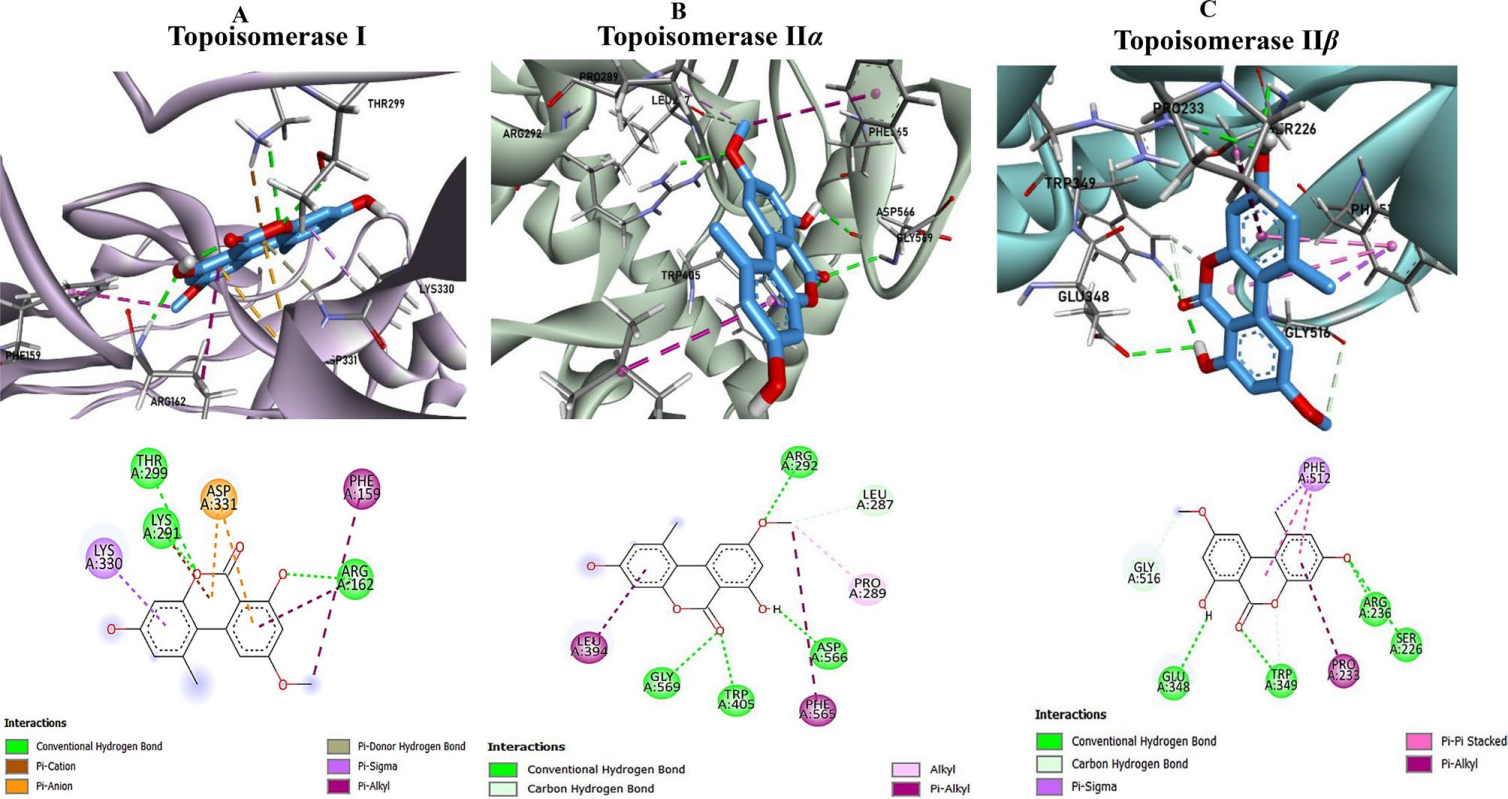
The three-dimensional (3D) (Upper panel) and two-dimensional (2D) (Lower panel) of Topoisomerase I (**A**) Topoisomerase Ii*α* (**B**) and Topoisomerase II*β* (**C**) sites docked with the AME.

The AME exhibited a binding affinity of –7.3 kcal/mol with Topoisomerase I. As shown in Fig. 8, key interactions included conventional hydrogen bonds with ARG162, LYS291, and THR299; a π–donor hydrogen bond and dual π–anion interactions with ASP331; a π–sigma interaction with LYS330; and π–alkyl contacts involving PHE159 and ARG162. At Topoisomerase II*α*, the binding affinity was –8.0 kcal/mol. Notably, AME formed conventional hydrogen bonds with ARG292, TRP405, ASP566, and GLY569, a carbon hydrogen bond with LEU287, an alkyl bond with PRO289, and π–alkyl interactions with LEU394 and PHE565, as depicted at Fig. 8.

The strongest binding was observed at Topoisomerase II*β*, with an affinity of –8.3 kcal/mol. Interaction mapping (Fig. 8) revealed conventional hydrogen bonds with SER226, ARG236, GLU348, and TRP349; carbon hydrogen bonds with TRP349 and GLY516; two π–π stacking interactions and one π–sigma bond with PHE512; and a π–alkyl bond with PRO233.

## Discussion

The resistance of tumor cells to most of the chemotherapeutic drugs is one of the current challenges in cancers treatment, especially with the drugs overuse and misuse. Various mechanisms of tumor cells drug-resistance were emerged such as genetic mutations, epigenetic changes, and alterations in drug transport or metabolism^52^. Drug efflux by overexpressing the permeability glycoprotein (P-gp) is one of the most common multidrug drug resistance approach (MDR) in tumor cells that pump the drugs out of the cell, reducing their intracellular concentration and effectiveness^52^. The chemotherapeutic drugs of topoisomerases inhibitory activity are one of the most common prescribed drugs in cancer treatment, however, the availability and cytotoxicity of the current drugs are the common challenge, thus, probing for a novel fungal sources with higher AME concentration, evaluating their cytotoxicity, in addition to the molecular modeling analysis is the objective.

Twenty-four endophytic isolates of *S. nicolai* were recovered on different medium, for the first time, as revealed from the literature. So, this is first report describing the fungal endophytic profile of *S. nicolai,* that could open a new platform for exploring the different biotechnological insights of this plant, in addition to their fungal plethora, that could have unique biological activities. Interestingly, *S. nicolai* had a noticeable resistance to the common fungal pathogens except *Bipolaris oryzae* and *Phytophthora nicotianae* that causes leaf spot and blight^53,54^. The obvious resistance to common fungal diseases of this plant reveals the unique endophytic fungal flora and their positive physiological roles on maintenance the overall plant health. Among the twenty-four fungal isolates, *C. cladosporioides* EFBL-025 had the highest AME producing potency, as revealed from the TLC and HPLC analyses, compared to the authentic AME. The spectral finger print of the putative AME of *C. cladosporioides* was verified by the UV, and FT-IR analysis, with the typical maximum absorption peak at λ_365_ nm, of the authentic one (Cat.# HY-W013863), confirming the identity of sample as AME. Moreover, the structure of the purified AME of *C. cladosporioides* was resolved by LC/MS as 274.2 m/z, with molecular ion peaks at 256.12, 105.9, 87.9, 70.9, 69.9, 56.9, 42.9 and 41.83 m/z that structurally matched with the fragmentation pattern of authentic AME (Cat.# HY-W013863). Strikingly, this is the first report exploring a *Cladosporium cladosporioides,* as an endophyte of *S. nicolai*, with the metabolic potency to produce AME, with emphasizing their chemical structural identity. *Alternaria* species were reported to produce a plethora of mycotoxins with a host-specific and non-host-specific activity^55^. Among the non-host-specific *Alternaria* toxins are Alternaric acid, alternariol, alternariol 9-monomethyl ether (AME), brefeldin A, Altertoxins, tenuazonic acid and curvularin (as reviewed by^56^. So, for the first time outside of the genus *Alternaria*, *C. cladosporioides* had an obvious metabolic potency for conceivable production of AME as revealed from the HPLC and LC-MS/MS. Similarly, *Cladosporium* species, an endophyte of *T. mairei*, displayed a significant antifungal activity against the fungal pathogen; *Fusarium, Trichoderma, Neurospors, Stachybotrys, Curvularia,* and *Verticillium*^57^. Similarly, brefeldin A has been reported to be produced by *Alternaria* species^56^ and *Aspergillus clavatus*^58^ with a strong antibacterial and antifungal activity. So, the metabolic sharing of different fungi for production of the same metabolite has been reported frequently, for example for Taxol, Camptothecin, and Epothilone secondary metabolites^16,17,59–63^. The *Cladosporium* species have been reported as a highly resourceful fungal genus for natural products of diverse chemical structures and biological activities^64^. Overall, the metabolites derived from *Cladosporium* are diverse in skeletal chemical structures, ranging from polyketides, alkaloids, and terpenoids, with diverse biological activities such as cytotoxic, antifungal and enzyme-inhibitory activities^65–68^. Consistently, alternariol and alternariol 5-O-methyl ether were characterized from the *Cladosporium* sp., an endophyte of *Chrysosplenium carnosum*^69^.

The highest yield of AME was assessed at the 7^th^ days (700.1 μg/l) followed by 10^th^ day (504.3 μg/l) with a significant decreasing to the AME concentration by the 20^th^ days of incubation. The highest concentration of AME at the early of stationary phase reveals the implication and essentiality of this metabolite on the some vital biological process processes. The methanolic extract of *S. nicolai* has no effect on AME yield by *C. cladosporioides,* negating the presence of any plant-derived metabolites inducing the biosynthesis of AME. The yield of AME by *C. cladosporioides* was quietly stable along the tested 10 months, ensuring the relevant biosynthetic stability of AME by the fungus independent on external stimuli from plants*.* The yield of AME by *C. cladosporioides* was partially consistent to those of *Alternaria* sp grown in ground rice-corn steep liquor medium^70^. Similarly, the powerful antimicrobial activity of the extracted *Alternaria* sp AME was reported towards various pathogenic fungi *Magnaporthe oryzae*, and antinematodal activity against *Bursaphelenchus xylophilus* and *Caenorhabditis elegans*^51^.

The purified AME of *C. cladosporioides* had the highest activity against A549 (0.65 μg/ml) followed by HepG-2 (1.2 μg/ml), and MCF-7 cells (2.7 μg/ml), with selectivity index 28.2, 14.5, and 8.1 folds, respectively. The cytotoxic activity of AME *C. cladosporioides* being slightly higher than Taxol as positive control by 1.8 folds, ensuring the higher potency of AME to target the different key enzymes of essential cellular pathways, normalized to OEC control cells. From the kinetics of enzyme inhibition, the IC_50_ values of *C. cladosporioides* AME for topoisomerase I and II were lower than of Topotecan and Etoposide by 3.5 folds, proposing the significant dual affinity of AME to inhibit both topoisomerase I and II. Practically, the AME had a higher affinity as inhibitor to topoisomerase II than I by 27 %. Consistently, the EC50 value of AME towards the HepG-2 cells was 4.5 μg/ml, compared to Caco-2 cells (14.5 μg/ml)^13,71^. The AOH and AME were found to induce the ROS, and interact with DNA topoisomerases, generating single, double strand breaks, with subsequent suppressing the proliferation in mammalian cells, with cell cycle arrest at G2/M-phase^10,14,72–75^. The higher reactivity of AOH and AME could be due to their easily hydroxylation by CYP450 enzymes, as enzyme of phase 1, generating reactive catechols that subsequently forms reactive semiquinones and quinones resulting in ROS formation^76,77^. AME was reported to induce a stronger cytotoxic effects compared to AOH with significant decreases to the viability of the HepG2 cells^71^.

From the MCF-7 cell cycle for *C. cladosporioides* AME, the highest growth arrest was observed at the G0-G1 phase by 73.9 %, compared to control (56.03 %), suggesting the interference with the machinery of cell grows and protein synthesis prior to DNA replication. From the apoptosis analysis, the purified *C. cladosporioides* AME induce significantly induces the apoptosis processes of MCF-7 cells by 12 folds compared to the control cells. With the treatment of MCF-7 by AME, the late apoptosis was increased by 22.8 %, normalized to control cells. Consistently, the cytotoxicity of AOH and AME by inducing the apoptotic cell death through the mitochondrial pathway was reported^74,75,78^. The wound healing activity of the AME-treated MCF-7 cells was 84.6%, i.e the cellular healing activity was reduced by 12.5 %, compared to the control cells after 48h.

*In vitro* assays against *β*-tubulin and topoisomerases I and II revealed significant inhibitory activity by AME, so, the binding interactions were elucidated by the molecular docking. The *in vitro* assays revealed a significant inhibitory activity against *β*-tubulin and topoisomerases I and II, suggesting that AME effectively interferes with critical cellular targets involved in cell division and DNA replication^79^. For the tubulin targets, the AME exhibits differential binding across three sites. Although its affinity at the β-tubulin taxane site (-7.1 kcal/mol) was lower than that of Taxol, the ligand showed an improved affinities at the colchicine (-8.2 kcal/mol) and vinca (-8.3 kcal/mol) sites, suggesting distracting of the microtubule dynamics, potentially leading to effective microtubule destabilization^80–84^. The diverse non-covalent interactions, support a stable binding conformation that correlates with the *in vitro* inhibitory activity. The docking results provided further insights into the molecular interactions of AME with both Topoisomerase I and II isoforms. The binding affinities (-7.3 to -8.3 kcal/mol) and the extensive networks of hydrogen bonds and hydrophobic interactions observed in these complexes suggest that AME can effectively disrupt the enzymatic activity required for DNA replication and repair. This inhibition is consistent with the significant activity observed *in vitro*, thereby reinforcing the potential of AME as a dual-target anticancer agent.

In conclusion, *C. cladosporioides* was recovered for the first time as an endophyte of *Strelitzia nicolai*, morphologically and molecularly confirmed based on its ITS sequence with accession # PX463714, as a potential AME producer. The chemical identity of AME of *C. cladosporioides* was committed from the FT-IR, HPLC and LC-MS/MS analyses. The purified AME of *C. cladosporioides* had a significant activity against A549, HepG-2, and MCF-7 cells, with selectivity indices 28.2, 14.5, and 8.1 folds, respectively, compared to OEC cells. The AME of *C. cladosporioides* had a strong activity against the Topoisomerase I, and II, and tubulin polymerization, inducing the apoptosis process by about 12 folds, halting the cell cycle at the G0-G1 phase. From the molecular docking analysis, AME had lower binding energy for topoisomerase II, than I, ensuring the *in vitro* analysis, compared to topotecan and Etoposide. So, with further experimental, computational analyses, AME could be a novel candidate with higher cytotoxic activity via inhibiting the mitotic machinery and DNA synthesis of the tumor cells.

## Supplementary Information

Supplementary Information 1.

Supplementary Information 2.

## Data Availability

All the data are provided in the manuscript. The ITS sequence of *C. cladosporioides,* an endophyte of *S. Nicolai,* was deposited to Genbank with accession # PX463714 ( [https://submit.ncbi.nlm.nih.gov/subs/?search=SUB15719247).
