# Supplementary material for: Cladosporium cladosporioides, endophyte of Strelitzia nicolai, as a new producer of Alternariol monomethyl ether with a potential cytotoxic activity
Source: Sci Rep. 2026 Jan 12;16:1633. doi: 10.1038/s41598-025-33343-6 (PMC12800168; doi:10.1038/s41598-025-33343-6)
Supplement: Supplementary file 1 — Supplementary Information 1. [file 41598_2025_33343_MOESM1_ESM.pptx]

## Slide 1
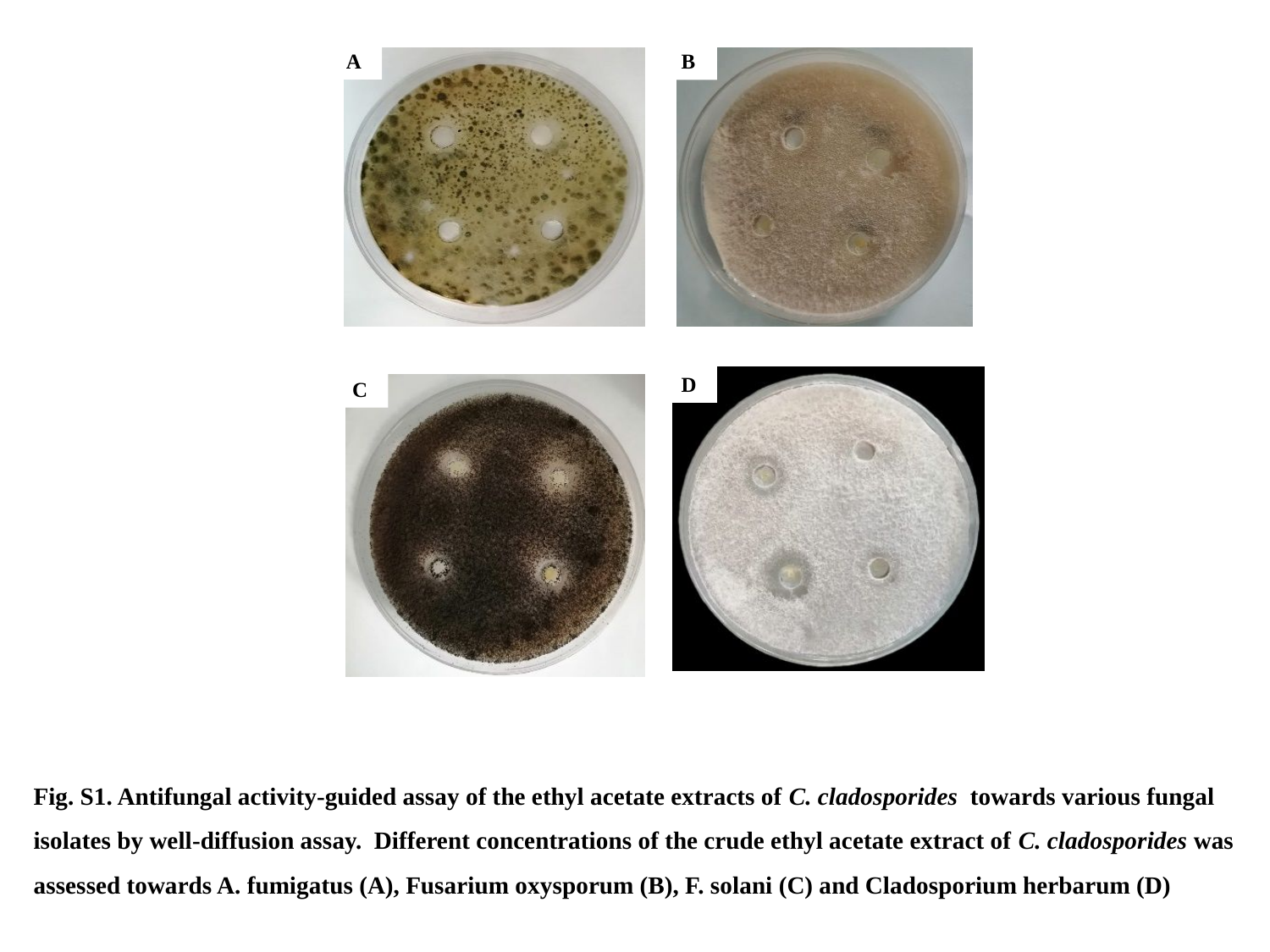

A
B
D
C
Fig. S1. Antifungal activity-guided assay of the ethyl acetate extracts of C. cladosporides towards various fungal isolates by well-diffusion assay. Different concentrations of the crude ethyl acetate extract of C. cladosporides was assessed towards A. fumigatus (A), Fusarium oxysporum (B), F. solani (C) and Cladosporium herbarum (D)
