# Supplementary material for: Cladosporium cladosporioides, endophyte of Strelitzia nicolai, as a new producer of Alternariol monomethyl ether with a potential cytotoxic activity
Source: Sci Rep. 2026 Jan 12;16:1633. doi: 10.1038/s41598-025-33343-6 (PMC12800168; doi:10.1038/s41598-025-33343-6)
Supplement: Supplementary file 2 — Supplementary Information 2. [file 41598_2025_33343_MOESM2_ESM.docx]

Table S1: Screening for the AME producing endophytic fungal isolates inhabiting *Strelitzia nicolai*

|  | Fungal isolate | TLC signs | AME yield by TLC (μg/l) | AME yield by HPLC  (μg/l) |
| --- | --- | --- | --- | --- |
| 1 | *Aspergillus fumigatus* 1 | - | - | - |
| 2 | *Aspergillus awamori* | - | - | - |
| 3 | *A. tamarii* | - | - | - |
| 4 | *A. flavipes* | - | - | - |
| 5 | *Fusarium oxysporum* 1 | - | - | - |
| 6 | *Fusarium oxysporum* 2 | - | - | - |
| 7 | *Fusarium solani* 1 | - | - | - |
| 8 | *Fusarium solani* 2 | - | - | - |
| 9 | *A. flavus* | - | - | - |
| 10 | *Drechslera* sp | + | 135 | 150 |
| 11 | *Alternaria alternata* | ++ | 215 | 202 |
| 12 | *A. nidulans* | - | - | - |
| 13 | *A. flavus* | - | - | - |
| 14 | *Penicillium italicum* | - | - | - |
| 15 | *A. oryzae* | - | - | - |
| 16 | *Humicola* sp | - | - | - |
| 17 | *Cladosporium cladosporides*  *EFBL-025* | +++ | 420 | 450 |
| 18 | *Fusarium sp* | - | - | - |
| 19 | *Cladosporium cladosporides* | ++ | 218 | 240 |
| 20 | *cladosporium herbarum* | - | - | - |
| 21 | *Fusarium sp* | - | - | - |
| 22 | *Alternaria solani* | ++ | - | - |
| 23 | *A. versicolor* | - | - | - |
| 24 | *Alternaria triticina* | ++ | - | - |

**Table S2. Target proteins, the corresponding grid coordinates, and amino acid residues of the active sites**

| Target protein | PDB ID | Grid box coordinates | | Amino acid residues of the active site |  |
| --- | --- | --- | --- | --- | --- |
|  |  | **Centers (x, y, z)** | **Sizes (x, y, z)** |  |  |
| *β*-Tubulin-Taxane Site | **1JFF** | **3.40717, -17.1234, 10.2263** | **23.8119, 19.6994, 24.2017** | VAL22, ASP25, GLU26, CYS210, LEU214, LEU216, ASP223, HIS226, LEU227, ALA230, SER233, GLY 234, PHE269, ALA270, PRO271, LEU272, THR273, SER274, ARG275, GLN278, GLN279, TYR280, ARG281, ALA282, LEU283, GLU287, LEU288, GLN290, GLN291, MET299, PRO357, ARG358, GLY359, LEU360, LYS361 | |
| *β*-Tubulin-Colchicine Site | **1SA0** | **119.501, 88.2613, 6.00524** | **20.5064, 22.7587, 25.1832** | ARG1, GLU2, GLU44, ARG45, ASN47, VAL48, ASP127, CYS128, GLN130, GLN133, ASN164, PHE166, TYR199, VAL235, THR236, CYS238, LEU239, PRO242, GLY243, GLN244, LEU245, ASN246, ALA247, ASP248, LEU249, LYS251, LEU252, ASN255, MET256, THR303, VAL304, ALA305, ALA306, VAL307, ARG309, GLY310, ARG311, MET312, MET314, VAL317, ASP318, MET321, LEU322, GLN325, ASN338, ASN339, VAL340, LYS341, THR342, ALA343, VAL344, CYS345, ASP346, ILE359 | |
| *αβ*-Tubulin-Vinca Site | **7Z7D** | **12.2814, 44.7283, 10.5138** | **44.5519, 39.6615, 38.9723** | **Chain B (*β*-tubulin):**  ALA9, GLY10, GLN11, CYS12, GLY13, GLN15, ILE16, ASP67, GLU69, PRO70, GLY71, THR72, ASP74, SER75, GLN94, SER95, GLY 96, ALA97, GLY98, ASN99, ASN100, LYS103, THR107, GLU108, SER138, GLY140, GLY141, GLY142, THR143, GLY144, SER145, VAL169, PRO171, SER172, PRO173, LYS174, VAL175, SER176, ASP177, THR178, VAL179, VAL180, GLU181, PRO182, ASN204, LEU207, TYR208, PHE212, THR218, THR219, PRO220, THR221, TYR222, LEU225, ASN226, VAL229, ILE380, GLN383, MET387, HIS395, TRP396, TYR397, THR398, GLY399, GLU400  **Chain C (*α*-tubulin):**  MET430, ARG431, GLU432, CYS433, GLY474, ASP475, ASP476, SER477, ASN479, THR480, ARG493, GLY560, LEU561, GLN562, GLY563, PHE564, LEU565, SER587, GLY591, LYS592, LYS593, SER594, LYS595, LEU596, LEU624, GLU625, HIS626, SER627, ASP628, ILE667, THR668, SER670, LEU671, PHE673, ASP674, GLY675, ALA676, LEU677, ASN678, VAL679, ASP680, LEU681, THR682, GLU683, GLN685, THR686, ASN687, VAL689, PRO692, ARG693, HIS695, PRO754, LYS755, VAL757, ASN758, ILE761, LYS765, PHE772, CYS776, PRO777, THR778, GLY779, PHE780, LYS781, VAL782, GLY783, ILE784 | |
| Topoisomerase I | **1EJ9** | **7.7356, -4.32619, 35.3245** | **43.5626, 54.6309, 42.7335** | ARG8, TYR9, PRO10, GLU11, GLY12, ILE13, LYS14, VAL24, PHE25, ALA26, PRO27, PRO28, TYR29, GLU30, PRO31, LEU32, PRO33, GLU53, THR56, PHE57, LYS60, MET61, HIS64, TYR66, TYR106, PHE107, GLN110, THR111, ARG114, LYS115, GLN116, MET117, LYS122, ILE125, LYS126, GLU128, ASN129, GLU130, LEU132, LEU133, ARG147, ALA149, ASN150, PHE151, LYS152, ILE153, GLU154, PRO155, PRO156, GLY157, LEU158, PHE159, ARG160, GLY161, ARG162, HIS165, LYS172, ARG174, THR209, TRP214, GLU216, GLN219, SER221, ILE222, LYS223, TYR224, ILE225, MET226, LEU227, ASN228, PRO229, SER230, ARG232, ILE233, LYS234, GLY235, GLU236, LYS237, ASP238, GLN240, LYS241, THR244, ALA245, ARG247, LEU283, ALA284, LEU285, ARG286, ALA287, GLY288, ASN289, LYS291, GLU295, THR296, ALA297, THR299, VAL300, GLY301, CYS302, SER304, LEU305, PHE327, GLY329, LYS330, ASP331, SER332, ILE333, TYR335, ASN337, GLN376, GLU380, GLY381, THR383, ALA384, LYS385, VAL386, ARG388, THR389, ILE426, LEU427, CYS428, ASN429, HIS430, GLN431, |  |
| Topoisomerase II*α* | **5GWK** | **20.0664, -40.8348, -40.5204** | **68.3166, 66.007, 85.3967** | GLU26, GLY27, ASP28, SER29, ALA30, LYS31, THR32, LEU33, VAL35, SER36, LEU38, GLY39, VAL40, VAL41, ARG43, ASP44, TYR46, GLY47, VAL48, PHE49, PRO50, LEU51, ARG52, GLYS53, LYS54, ILE55, LEU56, ASN57, ALA61, SER62, LYS64, GLN65, GLU68, ASN69, ALA70, GLU71, ASN74, ASP106, GLN107, ASP108, GLN109, ASP110, SER112, HIS113, ILE114, LYS115, LEU117, THR140, PRO141, ILE142, LYS144, SER146, ASN148, LYS149, GLN150, GLU151, TYR155, SER156, LEY157, PRO158, PHE160, GLU161, GLU162, TRP163, LYS164, SER165, HIS170, LYS171, LYS174, VAL175, LYS176, TYR177, TYR178, LYS179, GLY180, LEU181, GLY182, THR183, SER184, THR185, SER186, LYS187, GLU188, LYS190, GLU191, TYR192, ALA217, PHE218,SER219, LYS220, ILE223, ARG226, TRP229, THR231, PHE233, MET234, GLU235, ARG237, ARG238, ARG240, LYS241, LEU242, GLY244, LEU245, PRO246, GLU247, ASP248, TYR249, LEU250, TYR251, ASN265, LYS266, GLU267, LEU268, ILE269, LEU270, PHE271, SER272, ASN273, SER274, ASP275, GLU277, ARG278, SER279, ILE280, PRO281, SER282, ASP285, GLY286, LEU287, LYS288, PRO289, GLY290, GLN291, ARG292, LYS293, PHE296, PHE299, LYS300, ARG301, ASN302, ASP303, LYS304, ARG305, GLU306, VAL307, LYS308, ALA310, GLN311, SER315, ALA317, GLU318, MET319, SER321, TYR322, HIS323, HIS324, GLY325, GLU326, MET327, SER328, LEU329, MET330, MET331, THR332, ILE334, ASN335, LEU336, GLN338, ASN339, PHE340, VAL341, GLY342, SER343, ASN344, ASN345, LEU346, ASN347, LEU348, GLN350, PRO351, ILE352, GLY353, GLN354, PHE355, GLY356, THR357, ARG358, LEU359, HIS360, GLY361, GLY362, LYS363, ASP364, SER365, ALA366, SER367, PRO368, ARG369, TYR370, ILE371, PHE372, MWT374, LEU375, SER376, SER377, LEU378, ARG380, PRO385, LYS386, ASP388, HIS389, LEU391, LYS392, PHE393, LEU394, TYR395, ASP396, ASP397, ASN398, GLN399, ARG400, VAL401, GLU402, PRO403, GLU404, TRP405, TYR406, MET412, VAL413, ASN416, GLY417, ALA418, GLU419, GLY420, ILE421, GLY422, THR423, GLY424, TRP425, SER426, CYS427, LYS428, ILE429, PRO430, ASN431, TYR457, LYS458, ASN459, LYS461, ILE474, SER489, GLU490, VAL493, ARG494, TRP496, THR497, GLN498, THR499, LYS501, GLU502, GLN503, ARG520, GLU521, HIS523, THR524, ASP525, THR526, THR527, VAL528, LEU557, CYS559, PHE565, ASP566, HIS567, VAL568, GLY569, CYS570, LEU571, LYS572, ARG595, TRP598, LEU599, MET602, LEU603, ALA605, GLU606, LYS609, LEU610, GLN613, ASP647, VAL649, LYS650, TRP652, LYS653, GLN656, GLN657, TYR664, ASP667, MET668, PRO669, TRP671, TYR672, THR674, GLU676, LYS677, GLU680, LEU681, ARG683, LEU684, LYS688, GLU691, THR694, LEU695, LYS698, LEU703, GLU706, ASP707, LEU715, VAL718, GLU719, LYS721, GLU722, LYS723, GLN724 |  |
| Topoisomerase II*β* | **3QX3** | **30.1674, 110.044, 58.4627** | **76.3302, 91.1671, 65.9729** | ILE3, PRO4, LYS5, ASP7, ASN10, GLU26, GLY27, ASP28, SER29, ALA30, LYS31, SER32, LEU33, ALA34, VAL35, SER36, GLY39, VAL40, ILE41, ARG43, ASP44, PHE49, PRO50, LEU51, ARG52, GLY53, LYS54, ILE55, LEU56, ASN57, ALA61, LYS64, GLN65, GLU68, ASN69, ALA70, GLU71, ASN74, MET104, THR105, ASP106, GLN107, ASP108, GLN109, ASP110, HIS113, ILE114, LEU117, PHE138, THR140, LYS141, GLU142, ALA143, LYS144, TYR146, PHE147, ALA171, PHE172, SER173, LYS174, ILE177, ARG180, TRP183, MET188, ARG191, ARG192, ARG195, LEU196, LEU212, PHE215, SER116, ASN217, SER218, ASP219, ASN220, ASP229, GLY230, PHE231, LYS232, PRO233, GLY234, GLN235, ARG236, LYS237, PHE240, PHE243, LYS244, ARG245, ASN246, ASP247, LYS248, ARG249, GLU250, VAL251, LYS252, ALA254, GLN255, LEU256, GLY258, SER259, ALA261, GLU262, MET263, ALA265, TYR266, HIS267, HIS268, GLY269, GLU270, GLN271, ALA272, LEU273, MET274, MET275, THR 276VAL278, ASN279, LEU280, GLN282, ASN283, PHE284, VAL285,GLY286, SER287, ASN288, ASN289, ILE290, ASN291, LEU292, GLN294, PRO295, ILE296, GLY297, GLN298, PHE299, GLY300, THT301, ARG302, LEU303, HIS304, GLY305, GLY306, LYS307, ASP308, ALA309, ALA310, SER311, PRO312, ARG313, TYR314, ILE315, PHE316, LEU319, SER320, LEU322, ARG324, ALA329, VAL330, ASP332, ASN333, LEU335, LYS336, PHE337, ARG344, VAL345, GLU346, PRO347, GLU348, TRP349, TYR350, MET356, VAL357, ASN360, GLY361, ALA362, GLU363, GLY364, ILE365, GLY366, THR367, GLY368, TRP369, ALA370, LYS372, LEU373, PRO374, ASN375, TYR401, TRP440, THR441, GLN442, VAL443, LYS445, GLU446, GLN447, GLU450, PRO451, LEU453, ASN454, GLY455, THR456, ASP457, LYS458, ASP465, TYR466, LYS467, GLU468, TYR469, HIS470, THR471, ASP472, THR473, THR474, LEU504, PHE512, ASP513, HIS514, MET515, GLY516, CYS517, LEU518, TRP545, LEU546, MET549, LEU550, GLU553, LYS556, LEU557, GLN560, ASP594, VAL596, LYS597, TRP599, LYS600, GLN603, GLU604, ASN609, TYR610, ASN613, MET614, SER615, TRP617, SER618, LYS623, GLU626, LEU627, GLN630, LYS634, GLU637, LEU661, VAL664, GLU665, GLN667, GLU668, ARG669, ASP671 |  |
